# The Influence of Different Light Spectra on Broiler Chicken Endocrine Systems and Productivity

**DOI:** 10.3390/ani15213209

**Published:** 2025-11-04

**Authors:** Lenuța Galan, Gheorghe Solcan, Carmen Solcan

**Affiliations:** 1 Preclinics Department, Faculty of Veterinary Medicine, Ion Ionescu de la Brad Iași University of Life Sciences, 8 M. Sadoveanu Alley, 700489 Iași, Romania; lenuta.galan@iuls.ro (L.G.); carmen.solcan@iuls.ro (C.S.); 2 Clinics Department, Faculty of Veterinary Medicine, Ion Ionescu de la Brad Iași University of Life Sciences, 8 M. Sadoveanu Alley, 700489 Iași, Romania

**Keywords:** poultry management, monochromatic light, muscle development, somatotropic axis, hypothalamic-pituitary-gonadal axis, hypothalamic-pituitary-thyroid axis, hypothalamic-pituitary-adrenal axis

## Abstract

**Simple Summary:**

Light plays a crucial role in regulating physiological processes in broiler chickens. It not only helps birds see but also reaches deep into their brains, influencing important body functions. In broiler chickens, different LED light colors (e.g., green and blue) can affect the hormone systems that control growth, metabolism, and stress. Green light stimulates early muscle development by increasing growth-related hormones, while blue light supports later muscle formation and helps maintain oxidative balance. Light also affects the hormone melatonin, which regulates daily rhythms and influences how the body responds to light. Changes in day length and exposure to artificial light at night can impact hormone activity, growth, and stress responses. Understanding how light affects the bird’s body and applying smart lighting strategies can help improve broiler health, growth, and welfare.

**Abstract:**

In birds, light can penetrate the cranial bones and reach deep brain regions, where non-visual photoreceptors, especially in the hypothalamus, detect spectral and photoperiodic cues. Alongside retinal photoreception, deep-brain light sensing contributes to circadian entrainment and regulates melatonin secretion by the pineal gland. These light-driven pathways modulate endocrine activity, playing a key role in muscle development. This review explores how monochromatic light-emitting diode (LED) illumination, particularly green and blue wavelengths, affects the somatotropic axis (growth hormone-releasing hormone [GHRH]-growth hormone [GH]-insulin-like growth factor 1 [IGF-1]), the gonadal axis (gonadotropin-releasing hormone [GnRH]-luteinizing hormone [LH]/follicle-stimulating hormone [FSH]-sex steroids [testosterone, estrogen, progesterone]), the thyroid axis (thyrotropin-releasing hormone [TRH]-thyroid-stimulating hormone [TSH]-thyroxine [T_4_]/triiodothyronine [T_3_]), and the hypothalamic-pituitary-adrenal (HPA) axis (corticotropin-releasing hormone [CRH]-adrenocorticotropic hormone [ACTH]-corticosterone). Green light enhances early-stage muscle growth via GHRH and IGF-1 upregulation, while blue light supports later myogenic activity and oxidative balance. Light schedules also influence melatonin dynamics, which in turn modulate endocrine axis responsiveness to photic cues. Furthermore, variations in photoperiod and exposure to artificial lights at night (ALAN) affect thyroid activity and HPA axis reactivity, influencing metabolism, thermoregulation, and stress resilience. Together, ocular and intracranial photoreception form a complex network that links environmental light to hormonal regulation and muscle growth. These insights support the strategic use of LED lighting to optimize broiler performance and welfare.

## 1. Introduction

In contemporary poultry production systems, lighting represents a critical environmental factor that directly influences broiler growth performance and physiological development. Among the key parameters involved, the photoperiod, light intensity, and spectral compositions have been identified as major modulators of physiological responses and behavioral patterns in broiler chickens.

Optimizing lighting strategies in broiler housing has proven to be essential for enhancing not only production efficiency but also bird health status and overall welfare. In this context, the implementation of light-emitting diode (LED) technology has gained increasing attention due to its numerous advantages, including extended operational lifespan and improved energy efficiency [1], but also due to the fact that it positively influences behavior, resulting in higher quality meat with a better texture [2]. Previous studies on broiler production systems have demonstrated that the use of monochromatic LED lighting, particularly within the green and blue spectra, is associated with beneficial effects on growth performance [3] and muscle development [4,5,6,7,8,9,10,11,12,13].

Exposure to these wavelengths increases serum melatonin [14] and plasma testosterone levels and stimulates myofibril proliferation, thereby contributing to increased muscle mass and body weight [15,16]. Notably, green light has proven particularly effective in promoting early-stage muscle development, while blue light exerts a more pronounced stimulatory effect during the later stages [13]. Moreover, green monochromatic light has been reported to accelerate the myogenic activity of skeletal satellite cells [17,18], whereas blue light contributes to improved meat quality by enhancing broiler antioxidant capacity [8]. Consequently, it has been hypothesized that monochromatic light may promote growth and development through hypothalamic GHRH expression modulation in broiler chickens [19]. The combined application of green and blue LED lighting has been shown to exert synergistic effects on productive performance, resulting in improved feed conversion efficiency and higher carcass yield compared with the use of individual monochromatic lights or conventional white light sources [16,20].

The underlying molecular mechanisms responsible for these effects involve an upregulation of growth hormone receptor expression, as well as the stimulation of satellite cell proliferation within skeletal muscle, both of which are critical processes for muscle development [21,22]. The duration and timing of light exposure are critically important. The common use of broad-spectrum LED lighting at nighttime in poultry facilities leads to the continuous stimulation of avian photoreceptors, interfering with the normal hormonal rhythms that depend on periods of darkness. Proper light exposure management through regulating its intensity, duration, and spectral composition is essential in reducing the endocrine-disrupting effects of artificial light at night (ALAN) in broiler chickens [23,24,25,26].

Integrating internal physiological states with external cues, the endocrine system is especially susceptible and responsive to environmental variations. Hormonal levels fluctuate according to light and dark cycles, in turn impacting metabolic, immune, and reproductive functions [27,28]. Adjusting light spectra in managing the broiler rearing environment represents an effective strategy for enhancing growth performance. The significance of this approach is supported by evidence indicating that the use of green and blue monochromatic lighting does not exert adverse effects on blood biochemical parameters or on the health status of internal organs [15,16]. Controlled manipulation of the light environment may therefore serve as a valuable tool for optimizing productive performance and improving the overall efficiency of modern poultry production systems.

**Figure 1:**
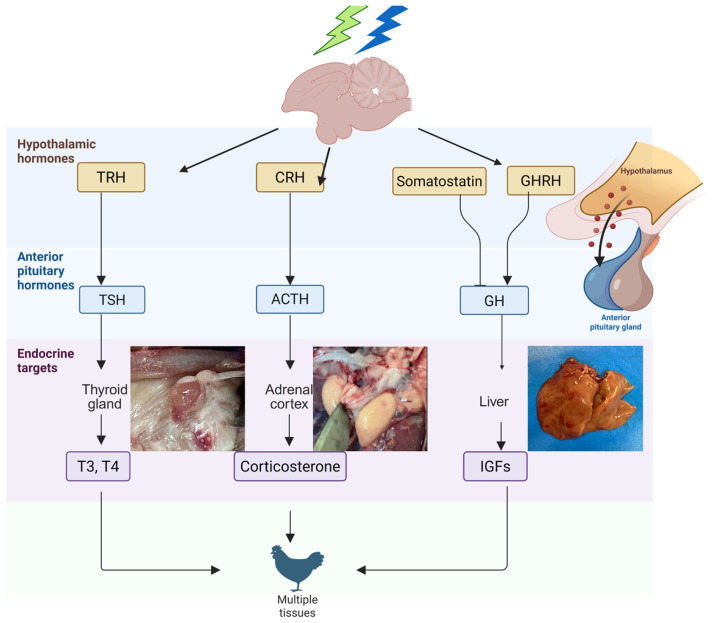
Presentation of the three axes, namely, somatotropic, hypothalamic-pituitary-thyroid, and hypothalamic-pituitary-adrenal, and the effect of light on them. Created in BioRender. https://BioRender.com/hruwpb3 (accessed on 20 October 2025).

The aim of this article is to explore how light, particularly various monochromatic wavelengths (green and blue), influences the activity of the pineal–hypothalamic axis in broiler chickens. We highlight how this influence extends to the functioning of endocrine glands such as the thyroid and adrenal glands, as well as to pectoral musculature development (Figure 1). Our analysis is based on studies demonstrating that variations in lighting conditions can modulate melatonin expression and its receptor activity, with direct effects on the secretion of GH, IGF-1, and other hormones essential for poultry performance [19,29,30]. Through this approach, we aim to provide an integrated perspective on the relationship between light stimuli and the endocrine responses of the avian organism.

## 2. Light Detection Pathways: Visual and Non-Visual Mechanisms in Physiological Regulation

Diurnal animals’ ability to perceive and integrate light-related information is essential for appropriate environmental adaptation, allowing them to adjust to both circadian and seasonal variations [31,32]. Light functions as a major environmental cue, significantly shaping animal behavior and playing a vital role in regulating physiological functions, productivity, and overall welfare across various animal and avian species [33,34,35,36,37]. Among its many physiological roles, light supports visual perception, modulates reproductive hormone secretion, and influences social interactions. In birds, the most notable physiological responses to light are associated with changes in photoperiod and light intensity, which are known to affect reproductive cycles, health status, and behavioral patterns [21,35,38].

**Figure 2:**
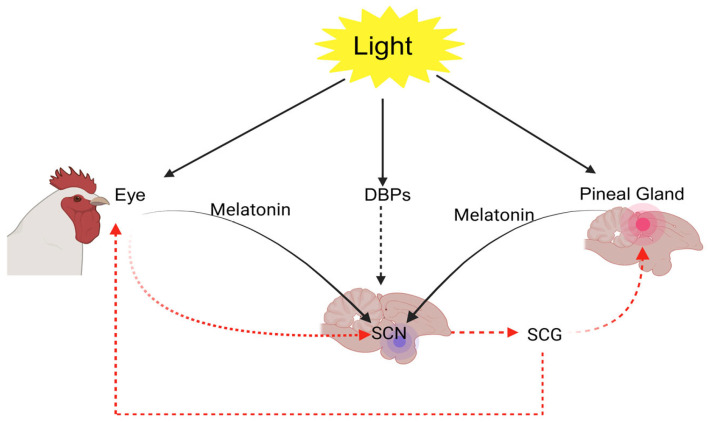
Interaction of oscillators in the pineal, SCN, and eyes coordinates circadian rhythms in birds. Action of light on the eye, the pineal, and deep-brain photoreceptors (DBPs) (→). Endocrine connections (melatonin). Neuronal connections (→). Superior cervical ganglia (SCG). Created in BioRender. https://BioRender.com/ro5xzf2 (accessed on 20 October 2025).

Poultry, in particular, possess a highly advanced visual system that plays a central role in mediating behaviors such as feeding, drinking, and social recognition [39]. Broiler chickens perceive light primarily through multiple photoreceptive sites, including the retina, pineal gland, and extraretinal photoreceptors [40,41,42] (Figure 2).

The chicken retina contains both rods, which facilitate vision under dim lighting, and cones. Notably, the retinal structure in poultry is among the most developed cone systems found in vertebrates. It includes five types of photoreceptor cones: four that are maximally sensitive to violet, blue, green, and red wavelengths of light [43,44], and one double cone that is thought to be involved in achromatic motion detection [16,42]. Beyond visual perception, light influences a wide spectrum of physiological and behavioral processes in poultry. The circadian energy homeostasis control is maintained via an intrinsic biological clock situated in the suprachiasmatic nuclei (SCN) of the hypothalamus. This clock becomes synchronized with environmental lighting through photic input from intrinsically photosensitive retinal ganglion cells, aligning physiological and behavioral rhythms with the light-dark cycle [45].

Light represents a powerful environmental cue for entraining the circadian system, although other factors such as feeding patterns can also modulate its signaling [45]. As a result, light exposure plays a vital role in shaping both growth and developmental trajectories in poultry. Responding to changes in photoperiod requires the coordinated function of three components: photoreceptors capable of detecting light, an internal timing mechanism that assesses day length, and an effector system that conveys photoperiodic information to the rest of the organism [46,47]. Numerous studies have shown that birds possess photoreceptors in various brain areas, including the pineal gland and septal regions of the telencephalon [48,49]. Birds’ extraretinal photoreceptors include several types of opsins. For instance, Opn4, Opsin 5, and vertebrate ancient opsin have been identified in the avian brain, where they respond to photoperiodic information and influence the onset and development of reproductive function. All three types of deep-brain photoreceptors (DBPs) appear to be involved in priming the neuroendocrine system to activate avian reproductive functions [46,47,50].

These opsins exhibit peak light sensitivity around 420, 480, and 490 nm, respectively [51,52,53,54]. For these deep-brain photoreceptors to be activated in non-mammalian vertebrates, light must pass through overlying tissues.

Consequently, the light spectrum that ultimately reaches these photoreceptors is largely shaped by the transmission characteristics of the intervening tissue layers [51,55]. As light passes through biological tissues, its properties are primarily altered by scattering, which favors the transmission of longer wavelengths over shorter ones. Additionally, the presence of light-absorbing molecules within the tissue further shapes the spectral profile of the transmitted light. Hemoglobin is the dominant absorber in this context, creating a spectral window that permits enhanced light transmission within the 460–540 nm range to reach internal photoreceptors [56,57].

When considering the impact of artificial light at night (ALAN), the spectral sensitivity of non-visual photoreceptors plays a key role in determining how vertebrate species perceive and respond to light stimuli. For a physiological or behavioral response to occur, the emitted spectrum of artificial light must coincide with the photoreceptor’s sensitivity range. If there is no such overlap, the light is unlikely to be perceived or to provoke any biological effects. Evidence of clock gene expression in both the mediobasal hypothalamus (MBH) and the suprachiasmatic nuclei (SCN) supports the hypothesis that birds’ photoperiodic timekeeping mechanism is located within the hypothalamus [58]. Alongside these insights, thyroid hormones have long been recognized as key regulators of the avian photoperiodic response [59].

Yoshimura et al. [60] demonstrated that in Japanese quail, the growth of gonads in response to day length is regulated by the light-stimulated conversion of the thyroid hormone thyroxine (T4) into its active form, triiodothyronine (T3), within the mediobasal hypothalamus (MBH). The enzyme responsible for this conversion, type 2 iodothyronine deiodinase (Dio2), is upregulated by light exposure. Increasing T3 levels in the brain replicates the gonadal growth normally triggered by long photoperiods, whereas blocking Dio2 prevents this development. Therefore, the light-induced activation of Dio2 and the resulting production of T3 in the MBH represent a key mechanism underlying photoperiodic reproductive function control in birds.

## 3. Interactions Between the Endocrine System and the Circadian Clock in Avian Physiology

Among vertebrates, circadian rhythms are predominantly governed by intracellular transcriptional and translational feedback loops involving core clock genes. These circadian processes play a critical role in the regulatory mechanism of reproductive functions in Japanese quails, as the reproductive system represents a primary endocrine function affected by light-driven rhythmic activity [61]. In contrast to mammals, the per1 gene (Period Circadian Regulator 1) is not expressed in birds, and avian cryptochromes, encoded by cry genes, may also possess light-sensing capabilities [62]. The principal molecular clock mechanism is supplemented by several auxiliary feedback loops that support physiological function regulation. One of these auxiliary loops establishes a connection between metabolic processes and the central circadian clock through metabolic sensors that interact with the core clock gene bmal1 (Basic Helix-Loop-Helix ARNT Like 1, also known as arntl). Another loop is linked to stress regulation; it modulates corticosteroid receptor activity via core clock gene interactions throughout the day [63]. Moreover, both central and peripheral feedback loops are tightly integrated with the immune system in a bidirectional regulatory relationship [64,65,66].

Photic information is transmitted to the molecular clock via cyclic adenosine monophosphate (cAMP) response elements that influence core clock and immediate early gene expression [67,68]. The main circadian loop is also coupled with melatonin signaling, as it controls the expression of enzymes involved in melatonin synthesis [69], while melatonin itself feeds back to modulate bmal1 expression [70].

Endocrine-circadian crosstalk is further reflected in the rhythmic secretion patterns of several hormones, as well as in the reciprocal influence of endocrine pathways on circadian regulation. For example, corticosterone-the primary glucocorticoid secreted through the hypothalamic-pituitary-adrenal (HPA) axis-follows a clear circadian rhythm, with peak concentrations occurring just before the onset of daily activity [71]. This pattern is generally conserved among bird species, though seasonal modulation and geographic variation (such as at higher latitudes) can influence its expression [72,73,74]. In addition, sex steroids like testosterone and estradiol display distinct circadian rhythms [75,76] and can, in turn, exert regulatory effects on circadian system functioning [77,78].

## 4. Pineal Gland

In birds, the pineal gland (*epiphysis cerebri*) is located on the dorsal surface of the brain, positioned within a triangular region between the cerebral hemispheres and the cerebellums. During embryonic development, it originates as a neuroepithelium outgrowth from the dorsal region of the posterior diencephalon, specifically between the habenular nuclei and the posterior commissure. Throughout evolution, the pineal gland has undergone significant functional and structural changes: while it served both secretory and photoreceptive roles in species like fish and amphibians, it evolved into a solely neuroendocrine organ in mammals, receiving light input indirectly via neural pathways. These evolutionary adaptations were accompanied by modifications in the gland’s structural and neuronal organization. Among birds, there is notable interspecific variation in both the anatomy and physiological roles of the pineal gland [79,80,81,82,83]. The avian pineal gland typically consists of a distal expansion called the pineal vesicle, which lies against the skull and is attached to the dura mater, and a narrower proximal part-the pineal stalk-connecting it to the dorsal wall of the third ventricle. In domestic chickens (*Gallus domesticus*), the pineal gland measures approximately 1.8 mm in width and 2.8 mm in length [84].

The pineal gland maintains bidirectional communication with the brain via efferent and afferent neural pathways [84,85]. In many bird species, the pineal gland receives dense innervation, primarily through noradrenergic postganglionic sympathetic fibers that originate from the superior cervical ganglia (SCG). However, unlike passerine birds, galliform species exhibit only limited neural projections from the pineal gland to the brain. The avian pineal gland consists of photoreceptor and ependymal (interstitial) cells, as well as intrinsic neurons [86]. Exhibiting characteristics similar to photoreceptors, pinealocytes are modified sensory cells that possess reduced outer segments. In numerous avian species, these cells lack synaptic connections with the gland’s intrinsic neurons. Nonetheless, immunohistochemical evidence of light-sensitive pigments such as pinopsin, which shares structural similarities with rhodopsin, has been identified in the outer segments of pinealocytes, indicating their photoreception capacity [87]. Experimental studies have further demonstrated that melatonin production in isolated pineal cells is strongly regulated by light exposure [88,89]. Reflecting the neuroendocrine nature of the pineal gland, pinealocytes contain dense-core secretory vesicles located in the Golgi apparatus and along the basal processes. The neuronal population within the gland mainly comprises bipolar cells, although pseudounipolar neurons are also present in certain bird species. Electron and light microscopic studies have shown that the pineal gland parenchyma includes not only secretory cells but also immune cells such as lymphocytes, plasma cells, basophilic granulocytes and eosinophils. This lymphoid component forms what is termed ‘lympho-pineal tissue’, suggesting an immunologic role for the gland in addition to its endocrine function. The number of proliferating cells in the pineal parenchyma is significantly higher than in the interstitial tissue, implying that pineal products may influence postthymic T cell differentiation. During development, the pineal gland in birds shows significant morphological and cytological changes, with the number and size of follicles increasing during embryonic stages and cells localizing to the outer layers as development progresses. These features emphasize the pineal gland’s dual role in endocrine melatonin secretion and potential immunomodulation in broiler chicks [90,91,92].

The pineal gland is the primary site of melatonin synthesis, a neurohormone that plays a critical role in regulating the organism’s daily and seasonal rhythms. Its production is stimulated during darkness and suppressed by light exposure [93,94], a process governed by a multisynaptic neural pathway that links the pineal gland to environmental light cues via the retina. In the avian pineal gland, melatonin synthesis is regulated through three interrelated mechanisms: the presence of direct photoreceptors, including light-sensitive photopigments, an intrinsic circadian oscillator, and noradrenergic transmission [95]. Melatonin levels are influenced not only by exposure to light and darkness but also by additional external factors such as ambient temperature and thermal stress [96]. It is estimated that approximately 80 percent of the circulating melatonin in birds originates from the pineal gland [97], while the remaining amount is produced by the retina and other structures, including the skin, gastrointestinal tract, and immune cells [98]. Due to its lipophilic nature, melatonin is capable of rapidly diffusing into various tissues and organs via both the bloodstream and the cerebrospinal fluid. Melatonin receptors are found in the central nervous system, particularly in the hypothalamus, the suprachiasmatic nucleus (SCN), and the preoptic area, as well as in peripheral organs such as the liver, lungs, ovaries, spleen, and gastrointestinal tract [99,100].

**Figure 3:**
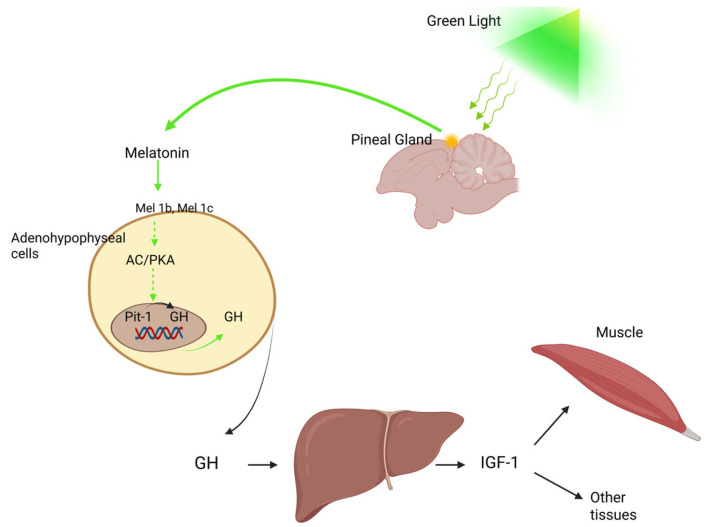
Green monochromatic light stimulates melatonin, which upregulates Pit-1 and GH in adenohypophyseal cells via Mel1b/Mel1c receptors. Elevated GH then promotes hepatic IGF-1 production, which drives muscle development in broiler chicks. Created in BioRender. https://BioRender.com/gl3c3os (accessed on 20 October 2025).

Monochromatic light has been shown to play a major role in regulating poultry growth and development [101,102]. More specifically, exposure to green monochromatic light through the action of melatonin enhances Pit-1 and GH mRNA expression and elevates GH levels in the adenohypophyseal cells of chicks, a process involving the melatonin membrane receptors Mel1b and Mel1c [103,104] (Figure 3).

Melatonin can translate environmental cues into endocrine responses, a function that enables the synchronization of animal physiology, metabolism, and behavior with optimal environmental conditions. Aligned with photoperiodic changes, melatonin plays a fundamental role in regulating neuroendocrine processes, contributing to the biological activity of virtually all cells [105]. It also influences growth and health status [106], as well as circulating hormone levels in poultry [107]. Melatonin is closely linked with central serotonin metabolism, and several of its physiological effects are mediated through serotonergic neurotransmission modulation. In chickens, melatonin has been shown to increase serotonin concentrations [108]. Furthermore, the central adrenergic pathway in birds, which regulates growth hormone (GH) secretion [109], is also modulated by melatonin. In addition to its essential role in regulating circadian rhythms, melatonin is involved in a wide range of physiological processes, including immune function [110], reproduction, thermoregulation, energy metabolism, and antioxidant defense. This molecule directly influences lymphocyte production and antibody synthesis [111], while also contributing to the neutralization of free radicals [111,112], thereby preventing oxidative stress. Its antioxidant properties [113] are also evident in the context of liver protection and adaptation to heat stress [114,115]. Moreover, melatonin mediates the effects of monochromatic light on broiler chicken growth and development [116].

## 5. Hypothalamus

The hypothalamus is a crucial brain region located beneath the thalamus and dorsally to the anterior part of the pituitary gland. It plays a central role in linking the nervous and endocrine systems [117], primarily through the synthesis and release of specific neurohormones known as releasing hormones and neurotransmitters, which stimulate pituitary hormone secretion. The hypothalamus also contributes to regulating essential physiological functions, including hunger and thirst, circadian rhythms and sleep, body temperature, and fatigue levels [118,119]. The hypothalamus plays a pivotal role during developmental stages, primarily by facilitating the effects of growth hormone (GH), which in turn impacts somatic development, body composition, lipid metabolism, physical performance, and agility. It also governs intermediary metabolism through the secretion of essential regulatory hormones, including growth hormone-releasing hormone (GHRH), somatostatin (SST), and ghrelin (GHRL), which collectively influence GH activity. Unlike in mammals, where hypothalamic subregions can be functionally distinguished using Nissl staining, avian hypothalamic structures cannot be as readily dissected for individual function [117]. Therefore, as many elements of its structure and regulation remain incompletely understood, the avian hypothalamus continues to be a key area of neuroendocrine research.

Recognized as a central neuroendocrine integrative hub, the hypothalamus plays a crucial role in maintaining homeostasis by regulating key physiological processes through major endocrine axis control. The activity of this regulatory center is influenced by melatonin, which exerts its effects on hypothalamic neurons through specific receptors (Mel1a, Mel1b, Mel1c) located in critical areas such as the suprachiasmatic nuclei (SCN), the preoptic area (POA), and other regions involved in thermoregulation, metabolic control, and feeding behavior [120].

Hormonal axes represent complex networks of interactions involving the hypothalamus, the anterior pituitary, and downstream somatic target tissues, playing a crucial role in regulating growth and development [121]. In vertebrates, four major endocrine axes influence these processes: the somatotropic, gonadal, corticotropic, and thyrotropic axes [122,123].

The somatotropic axis consists of growth hormone-releasing hormone (GHRH), growth hormone (GH), the GH receptor (GHR), insulin-like growth factors (IGFs), the type 1 IGF receptor (IGFR1), and IGF-binding proteins (IGFBPs). Through its actions, this axis promotes skeletal and muscle mass development, primarily by inhibiting apoptosis and reducing lipid accumulation in adipose tissue [124,125,126,127,128,129,130,131,132,133,134,135].

The hypothalamic-pituitary-gonadal (HPG) axis constitutes a central neuroendocrine system that tightly regulates growth and reproductive function in poultry. Within this regulatory network, gonadotropin-releasing hormone (GnRH) produced by the hypothalamus binds to specific receptors on pituitary gonadotropes, stimulating the synthesis and secretion of follicle-stimulating (FSH) and luteinizing hormones (LH). Once released into the bloodstream, FSH and LH act on the gonads to promote sex hormone production. These sex hormones, in turn, exert feedback control on the hypothalamus and pituitary, modulating the ongoing synthesis and release of GnRH, FSH, and LH, and thus maintaining precise reproductive activity regulation [136].

Within the corticotropic axis, the main hormone is corticosterone (CORT), which is secreted by the adrenal cortex under the influence of adrenocorticotropic hormone (ACTH), itself stimulated by corticotropin-releasing hormone (CRH) produced in the hypothalamus. The physiological effects of CORT are mediated through its binding to the glucocorticoid receptor (NR3C1) and its association with corticosteroid-binding globulin (CBG). The activation of this system leads to energy mobilization in response to stress, increased food intake, decreased feed conversion ratio (FCR), and enhanced lipogenesis [137,138,139,140,141,142,143,144].

The thyrotropic axis involves the hypothalamic production of thyrotropin-releasing hormone (TRH), which stimulates the secretion of thyroid-stimulating hormone (TSH) [145,146,147,148,149,150]. In turn, TSH promotes the release of thyroid hormones (THs) T4 and T3 [151,152,153], the activity of which is regulated locally in target tissues through deiodinases (DIOs), enzymes responsible for the conversion and inactivation of thyroid hormones [154,155].

## 6. Implications of Hormone Signaling in Muscle Tissue Development

In their mature form, skeletal muscle fibers are encased and structurally supported by three connective tissue layers: the endomysium, perimysium, and epimysium. This connective tissue consists of cellular elements and an extracellular matrix (ECM), which contains both fibrous and non-fibrous proteins, notably collagens and proteoglycans. Healthy muscle tissue is characterized by clearly delineated muscle fibers, distinct endomysial spaces separating individual fibers, and well-organized perimysial spaces between fiber bundles. The preservation of this spatial organization is essential for preventing degenerative changes in muscle fibers [156]. The pectoralis muscle primarily consists of type II glycolytic fibers, which rely on anaerobic metabolism. This metabolic pathway leads to the production of lactic acid, which is predominantly cleared from the muscle via the circulatory system and subsequently reconverted to glycogen in the liver [157]. Various growth factors play critical roles as either promoters or suppressors of myoblast and satellite cell proliferation and differentiation. Among the key regulators influencing muscle hyperplasia and hypertrophy are hepatocyte growth factor (HGF), fibroblast growth factor 2 (FGF2), insulin-like growth factor (IGF), transforming growth factor-beta (TGF-β), and myostatin [156].

Given the economic significance of skeletal muscle tissue, understanding the cellular mechanisms underlying muscle growth and regeneration in broiler chickens is essential. Muscle development predominantly occurs through satellite cell proliferation [158]. Multiple studies have shown that the regenerative activity of satellite cells, specifically their proliferation and differentiation, is strongly regulated by neural innervation, vascular supply, hormonal signals, nutritional status, and the degree of tissue injury [159,160,161,162,163]. Notably, satellite cells that remain in contact with the sarcolemma of intact myofibers display reduced sensitivity to mitogenic signals [164]. Under physiological conditions, satellite cells remain in a dormant state and do not undergo cell division [165]. Nevertheless, their proliferation can be activated either to replenish the satellite stem cell pool or initiate muscle tissue repair [166]. Once activated, these cells and their descendants are known as myoblasts [165,167], which are distinguished by the prompt expression of key myogenic transcription factors such as myoblast determination protein 1 (MyoD1) and myogenic factor 5 (MYF5) [168,169]. For muscle fiber development to occur, myoblasts must differentiate into contractile structures, a process that involves the formation of myotubes—elongated, multinucleated, post-mitotic cells resulting from the fusion of several myoblasts [158]. This fusion process requires the withdrawal of myoblasts from the cell cycle and the upregulation of myogenic regulatory factors (MRFs) [170,171,172]. Initial myotubes continue to develop as they fuse with additional myoblasts, resulting in the formation of mature myotubes [173]. In adult animals, the number of muscle fibers composed of mature myotubes remains generally stable, and muscle growth occurs via increases in the size of these existing fibers [174].

According to Orna Halevy [17], exposure to green and blue lighting promoted superior body and muscle development compared with red and white lighting, a result that was strongly associated with increased satellite cell proliferation in chickens. In broiler chicks, muscle fiber formation is mostly completed by the time of hatching. During embryonic development, myoblasts proliferate and differentiate into myotubes, determining the final number of muscle fibers present at hatch [156,175]. After hatching, muscle growth relies on the fusion of satellite cells with existing myotubes and the subsequent increase in the number of nuclei [176]. Additionally, enhanced protein synthesis is necessary to ensure that protein degradation does not exceed synthesis rates [156]. Rapid muscle growth in newly hatched chicks is supported by the high proliferation rate of satellite cells, their DNA synthesis, and fusion with myotubes [177]. However, this growth process is dependent on sufficient dietary energy. Chicks deprived of feed for 24 to 48 h post-hatch show significantly reduced muscle development compared with those fed immediately after hatching [177,178]. This period of intense muscle growth typically ends around three to four weeks of age, though growth continues at a slower pace beyond this point [179]. The immediate post-hatch phase is thus the most crucial window for muscle development in chicks.

Satellite cell activity is regulated by the expression of several key genes, particularly myogenic regulatory factors (MRFs) such as myogenic determination factor 1 (MyoD1) and myogenin. MyoD1 is expressed in activated and proliferating satellite cells and plays a vital role in directing their developmental pathway [180,181]. As satellite cells progress toward terminal differentiation, myogenin expression increases, which is essential for the completion of this process [180,182]. MRF expression can be negatively influenced by feed deprivation or restricted feeding regimens in chicks [183,184] and in satellite cell cultures [185,186], which, in turn, affects breast muscle development and growth.

Hormonal signaling plays a significant role in regulating myogenesis, and muscle tissue development is influenced by the function of the somatotropic [187] and thyrotropic hormonal axes. While most research in this field has been carried out on mammalian models, several studies have also investigated chickens. For instance, treating chicken satellite cells with human IGF-1 and IGF-2 stimulates DNA synthesis [188]. This finding indicates that IGFs promote muscle hypertrophy by activating satellite cells, which later fuse with existing myotubes. Conversely, IGF-binding proteins (IGFBPs) reduce these effects; by binding growth factors, they limit their availability and lead to lower DNA synthesis in vitro [189].

Thyroid hormones (THs) can also stimulate muscle growth. Their actions are, however, context-dependent and vary with developmental stage. Embryonic chicken thigh myoblasts cultured in vitro did not divide when exposed to T4; instead, they differentiated into myotubes and remained in this state longer than untreated cells [190]. This suggests that during embryonic development, T4 plays a key role in promoting myoblast differentiation. Nyoman Suthama et al. [191] investigated the effects of dietary T4 supplementation on growth performance and muscle protein metabolism in broiler chickens. Moderate doses (0.4–1.2 ppm) promoted feed efficiency, muscle mass deposition, and reduction in abdominal fat, with sex-related differences (females showed reduced fat accumulation, while males exhibited enhanced muscle protein synthesis). In contrast, higher doses (3.6 ppm) had negative effects, leading to body weight, muscle mass, and liver weight decreases, although protein metabolism was intensified in males. Therefore, moderate T4 administration may contribute to optimizing growth and improving carcass quality by stimulating protein synthesis and reducing fat accumulation. In young chickens, dietary supplementation with T3 (0.1 mg/kg) increased thigh muscle growth from hatch to six weeks. However, the same treatment suppressed growth between six and eight weeks [192]. These observations indicate that thyroid hormones are essential for proper muscle development in early life but, nevertheless, their positive effects decline as birds grow older. Altogether, these findings confirm that both the somatotropic and thyrotropic axes contribute to muscle development, with their impact strongly dependent on the developmental stage. Light is another external factor that interacts with these endocrine influences. Light color, in particular, has a marked effect on hormonal balance in broilers. Green or green-blue light increases plasma concentrations of T3 and T4 [193,194]; in contrast, exposure to blue light lowers these hormones, along with melatonin and corticosterone [195,196]. Overall, green and blue light are generally considered more beneficial than red or white light. They reduce stress levels and help maintain calmer behavior in broiler chickens [197,198].

## 7. The Somatotropic Axis

### 7.1. Hormonal Influence on Tissues

Somatotropic hormones play a crucial role in regulating muscle, bone, and fat tissue development. The hypothalamus secretes GHRH [126], a peptide that is part of the adenylate cyclase-activating polypeptide superfamily and highly conserved across vertebrate species [199]. GHRH acts by promoting growth hormone (GH) production in the somatotroph cells of the anterior pituitary. In contrast, hypothalamic somatostatin (SST) inhibits GH synthesis by binding to the somatostatin receptor 2 (SSTR2) located in the anterior pituitary, thereby counteracting GHRH’s stimulatory effects [200,201].

Growth hormone secreted by the pituitary gland serves as a central effector in the somatotropic axis. It is a relatively unstable and straightforward protein, characterized by a single intrachain disulfide bond and a molecular weight of approximately 22 kDa [202]. The liver serves as a primary target organ for GH, producing the key effector molecules IGF-1 and IGF-2 in response to its stimulation [132,133]. In addition to its role in the GH–IGF signaling axis, GH can also exert direct metabolic effects independent of IGFs. Both GH- and IGF-dependent pathways are crucial for promoting longitudinal bone growth and increasing skeletal muscle mass.

One of GH’s main functions is enhancing hepatic IGF production, though IGFs are also locally expressed in skeletal muscle and other tissues [203] (Figure 3). The anabolic effects of IGFs on bones and muscles are primarily mediated through cell proliferation stimulation and apoptosis inhibition, mechanisms triggered by their binding to the type 1 IGF receptor (IGFR1) [204]. Unlike mammals, which possess two IGF receptors, only one has been characterized so far for avian species [205]. Both IGF-1 and IGF-2 can interact with IGFR1, although the latter binds with lower affinity than the former [206]. A critical regulatory component of the IGF-IGFR1 interaction is the insulin-like growth factor binding protein (IGFBP) family. In mammals, this group consists of seven members (IGFBP1-IGFBP7), while six have been identified in avian species. Despite being highly conserved across vertebrates [135,207,208,209,210], IGFBP6 has not been detected in any bird species to date [211,212]. IGFBP7 structurally differs from the other members and exhibits low IGF affinity; however, it binds insulin efficiently and can inhibit insulin receptor activation [213]. IGFBPs synthesized in the liver circulate in the bloodstream and can modulate IGF activity by enhancing or inhibiting receptor binding, extending IGF half-life, or altering tissue-specific IGF effects [214,215]. In addition to their role in IGF regulation, IGFBPs can also function independently. For instance, IGFBP2 has been shown to promote apoptosis in the absence of IGFs [216,217], whereas IGFBP5 can stimulate osteogenic cell proliferation [218].

IGFBPs contribute to IGF signaling fine-tuning by interacting with other elements of the somatotropic axis, such as the insulin-like growth factor acid-labile subunit (IGFALS), a key modulator of IGF activity [219]. The formation of a ternary complex involving an IGF, IGFBP3, and IGFALS significantly prolongs the circulating half-life of IGFs in the plasma [220,221]. Although IGFALS’s function has not yet been confirmed in chickens, its strong structural and functional conservation in humans and rats [222,223] suggests a likely similar role in avian species. Like IGFs and IGFBPs, IGFALS is mainly synthesized in the liver, with its gene expression induced by GH signaling [224]. Furthermore, IGFBPs are subject to regulation by specific proteases [225,226], which serve as a mechanism for controlling IGF bioavailability. The proteolytic cleavage of IGFBPs lowers their affinities for IGFs, thus releasing the growth factors to bind their receptors and exert biological effects.

IGFBPs influence growth regulation by interacting with IGFs to either increase or decrease receptor binding affinity, prolong hormone stability, or modify tissue-specific actions [214,215]. For example, IGFBP1 inhibits protein synthesis within skeletal muscles [227], whereas IGFBP2 and IGFBP4 act to suppress long bone elongation [218,228]. In myoblast cultures, IGFBP5 stimulates cell proliferation when complexed with IGF-1 but inhibits it when bound to IGF-2, while IGFBP4 restricts myoblast proliferation only in the presence of IGF-1 [229]. Additionally, certain IGFBPs have signaling capabilities independent of IGF binding; IGFBP2 can independently modulate apoptosis [216,217], while IGFBP5 can promote bone cell proliferation without IGF involvement [218]. IGFBPs thus offer an additional regulatory mechanism within the IGF system and possess direct biological functions beyond IGF mediation.

### 7.2. Hormone-Mediated Regulatory Signaling

The somatotropic axis is activated when growth hormone-releasing hormone (GHRH) is synthesized and secreted from the hypothalamic paraventricular nucleus (PVN), where it binds to its specific receptor (GHRHR) located on somatotroph cells in the anterior pituitary [127,128,129]. GHRHR is a G protein-coupled receptor (GPCR) embedded in the cell membrane [230,231,232]. In chickens, GHRHR shows predominant expression in the pituitary gland, while only low levels are present in certain extrapituitary tissues, including the brain, pancreas, kidneys, and testes [233]. Likewise, in rats, GHRHR and its splice variants are chiefly localized in the pituitary, where they play a key role in regulating growth hormone synthesis and release [234]. Upon ligand binding, the receptor activates the stimulatory Gα subunit of the associated heterotrimeric G protein, which, in turn, triggers adenylate cyclase to convert ATP into cAMP [235]. cAMP functions as a secondary messenger that activates protein kinase A (PKA), leading to transcription factor CREB (cAMP response element-binding protein) phosphorylation [236,237]. Once phosphorylated, CREB binds to DNA and initiates growth hormone (GH) gene transcription, ultimately promoting GH synthesis and release from somatotrophs.

This synthesis can be inhibited by the action of hypothalamic somatostatin (SST), which binds to somatostatin receptor 2 (SSTR2) on pituitary cells, blocking the PKA-dependent activation of CREB, and thus, suppressing GH transcription [200,201]. Similar to GHRHR, SSTR2 is also a GPCR [238]. This receptor exists in two isoforms-SSTR2A and SSTR2B-generated via alternative splicing [239]. Both isoforms couple to a G protein composed of α, β, and γ subunits that upon activation, inhibit adenylate cyclase activity [240]. The reciprocal release of GHRH and SST from the hypothalamus generates rhythmic fluctuations in GH output, resulting in a pulsatile pattern of secretion into the bloodstream.

Xiaojing Qin [241] demonstrated that the light spectrum influences SST levels in both the hypothalamus and pituitary of chicks, with the highest concentrations observed under red light (RL) and the lowest under green light (GL), findings that are consistent with the results of Zhang et al. [19] and Yue et al. [103]. Green light stimulates hypothalamic GHRH expression and increases plasma GH secretion, whereas red light has the opposite effect. In the hypothalamus, SST regulates GH and GHRH, while in the pituitary gland, it modulates both SST and GH. These mechanisms are essential for chick growth and development under monochromatic light. The biological action of SST is mediated by five receptor subtypes, SSTR1–5. According to Bossis and Porter [200], selective SSTR2 agonists strongly suppress both basal and GHRH-stimulated GH release, even at low nanomolar concentrations, whereas selective SSTR5 agonists reduce GH secretion only under basal conditions.

GH promotes IGF-1 synthesis in hepatocytes by interacting with the growth hormone receptor (GHR), a class I cytokine receptor that activates signaling via the JAK2/STAT5 and Src/ERK pathways [242,243,244]. In chickens, GHR is expressed not only in the liver but also in muscle and lymphoid tissues [234,245]. Akt is capable of activating various downstream targets involved in cell growth pathways [246]. One important target is the mechanistic target of rapamycin (mTOR) [247]. However, this activation is indirect and requires tuberous sclerosis complex 2 (TSC2) phosphorylation [248,249], which allows mTOR to form a complex with RPTOR, a regulatory protein [250]. This assembly, known as mTOR complex 1 (mTORC1), plays a central role in promoting protein synthesis, lipid formation, and mitochondrial development [251,252]. These cellular processes are driven by mTORC1 through the activation of transcription factors and translational regulators such as sterol regulatory element-binding proteins (SREBPs) and S6 kinases (S6Ks) [253,254].

### 7.3. The Effect of Light on the Somatotropic Axis

Post-hatch growth in broiler chickens primarily results from muscle fiber hypertrophy. This cellular enlargement is regulated not only by endocrine signals but also by environmental factors such as light [7,194,255]. Light influences the somatotropic axis through mechanisms involving extraretinal photoreception (notably by the hypothalamus and pineal gland), as well as melatonin-GH/IGF-1 pathway regulation. In chickens, hypothalamic photoreceptors are capable of directly detecting light stimuli.

Experimental evidence has shown that muscle fiber size increases in response to specific light wavelengths, such as green light (GL) and blue light (BL), but at different developmental stages. More specifically, green light was associated with hypertrophy during the early growth phase (days 0–26), whereas blue light had a more pronounced effect in the later phase (days 27–49) [7]. These effects may reflect underlying cellular processes, such as satellite cell proliferation prior to 3 weeks of age, and cellular degeneration thereafter [7,18].

The early post-hatch period appears critical for satellite cell activity. According to Liu et al. [18], the first three days post-hatching may represent a pivotal window for mitotic activity, which peaks at day 3. Similarly, Halevy et al. [17,256] reported that exposure to green light either during incubation or immediately after hatching enhances satellite cell proliferation, particularly evident at postnatal day 5; however, data for other time points remain unclear.

Further studies have demonstrated that green light enhances multiple physiological and molecular parameters in broilers. For instance, GL significantly increased hepatic IGF-1 secretion [29], upregulated PCNA expression in the liver of young chicks, and stimulated skeletal satellite cell proliferation [17,257]. In addition, GL was associated with increased GHRH expression in the hypothalamus [19], enhanced muscle growth [7,18], and improved meat quality [8,258]. These effects appear to be synergistic with those of growth hormone (GH), which plays a central role in promoting overall growth and productivity in broilers. Consistent with these findings, Helva [259] observed that from days 14 to 42, broilers exposed to GL and BL exhibited higher body weight compared with those exposed to white light (WL). These observations aligned with Liu et al. [18], who reported increased growth rates under GL and BL between days 5 and 21. Interestingly, in Helva’s study, green light further enhanced body weight gain between days 35 and 42. Additionally, the highest plasma IGF-1 concentration was recorded on the day of hatching, followed by a gradual decline up to day 42. Notably, light color significantly influenced IGF-1 concentration only on day 7, when WL-exposed chicks showed higher IGF-1 levels compared with those under GL and BL. However, no significant effects of light color were observed on plasma GH concentrations between days 7 and 42.

The lighting program also plays a crucial role in broiler chicken development and overall performance, as adequate feed intake during the first days of life is essential for achieving optimal growth. For this reason, near-continuous lighting schedules during the first week of life have become a common strategy for encouraging early feeding [260]. Nevertheless, many studies have shown that in broilers, prolonged exposure to almost continuous light can negatively influence growth rate, feed intake, feed conversion efficiency, mortality, and welfare [261,262]. It is therefore reasonable to consider that these adverse effects may also appear during the first week of life, when such lighting regimes are typically applied [263].

Supporting these observations, Yang Yang et al. [264] reported that constant light exposure during the early stages of life induces fear-related behaviors in broiler chickens, accompanied by elevated corticosterone levels and reduced blood melatonin/5-HT concentrations. Moreover, early-life exposure to constant light disrupts the expression of genes involved in circadian clock regulation, oxidative stress response, and the BDNF/TrκB/ERK signaling pathway in the avian hippocampus.

On the other hand, providing longer dark periods of about four to six hours during the growing phase does not negatively affect broiler performance or welfare [263,265]. Furthermore, recent findings indicate that extending the dark period even further, up to twelve hours, may actually enhance growth performance and welfare [266].

Overall, light management remains a key component of commercial broiler production systems. Research has demonstrated that extending the photoperiod or applying intermittent lighting schedules can significantly improve productive parameters such as feed intake, body weight, and feed conversion ratio, compared with constant or nearly continuous light exposure [267].

## 8. Hypothalamic–Pituitary–Gonadal Axis

### 8.1. Hormonal Influence on Tissues

The hypothalamus acts as an integrative center, receiving signals from the external environment, the gonads, and the body to coordinate reproductive system development and maturation by regulating the production of gonadotropins from the pituitary gland, thereby promoting growth and maintaining gonadal function [268].

GnRH is the primary hypothalamic neuropeptide responsible for stimulating the synthesis and release of the gonadotropins, luteinizing hormone (LH) and follicle-stimulating hormone (FSH) from the anterior pituitary, which, in turn, stimulate follicular maturation and steroidogenesis, triggering ovulation in females [269]. In this way, GnRH serves as a key link between the nervous and endocrine systems in regulating avian reproduction [270].

Synthesized by the external thecal layer of small ovarian follicles, estradiol promotes reproductive tract development, as well as secondary sexual trait and reproductive behavior manifestations [269,271]. It also contributes to the negative feedback mechanisms acting on the hypothalamus and pituitary gland [272]. Additionally, estradiol enhances the hepatic production of key yolk precursors, shifts bone metabolism toward medullary formation rather than cortical growth, and increases calcitriol activity, thereby elevating blood calcium levels and ensuring calcium availability for eggshell formation [273]. In contrast, progesterone secreted by the granulosa cells of large follicles plays a crucial role in regulating ovulation [269,271,274].

Avian reproduction is governed by a dynamic balance between GnRH stimulation and GnIH inhibition. These two systems operate antagonistically but remain interconnected and self-regulating, enabling precise gonadotropin secretion adjustment according to internal and external conditions [270].

GnIH fibers form direct synapses with GnRH neurons, modulating their electrical activity and suppressing GnRH-I gene expression. Consequently, the central administration of GnIH rapidly decreases LH levels, suggesting that GnIH may inhibit gonadal development and maintenance, as well as sexual maturation in birds, by suppressing gonadotropin synthesis and release [275].

Melatonin coordinates the GnRH-GnIH balance: during long nights, increased melatonin enhances GnIH expression in the PVN, inhibiting GnRH neurons, reducing gonadotropin secretion, and causing gonadal regression [276]. In contrast, during long days, decreased nocturnal melatonin reduces GnIH and increases GnRH activity, supporting reproductive maturation [277]. In this way, melatonin acts as a neuroendocrine translator, converting photoperiodic signals into changes in the GnRH/GnIH ratio and controlling the transition between reproductive activity and inactivity.

The GnRH-GnIH balance is further influenced by gonadal steroid levels (providing negative feedback on GnRH secretion and positive GnIH expression regulation), social and stress signals (activating GnIH via the HPA axis), and thyroid hormones, which contribute to reactivating hypothalamic photosensitivity [60,270].

These multiple interactions enable the complex integration of hormonal, seasonal, and behavioral information, ensuring precise reproductive cycle synchronization with optimal environmental conditions.

### 8.2. Hormone-Mediated Regulatory Signaling

The activity of neurons secreting type I gonadotropin-releasing hormone (cGnRH-I) is a pivotal factor in regulating reproductive function in birds. It is modulated by a complex network of neurotransmitters, neuropeptides, and glial cells, among which the glutamatergic system plays a key excitatory role in the hypothalamo-pituitary-gonadal (HPG) axis. N-methyl-D-aspartate (NMDA) administration has been shown to promote luteinizing hormone (LH) release, though its effect on cGnRH-I neurons appears to be indirect [278].

Vasoactive intestinal peptide (VIP) represents another crucial neuromodulator involved in regulating prolactin secretion. In quail, VIP interacts with cGnRH-I neurons and may indirectly influence their activity according to photoperiodic cues [279]. The proximity of VIP and cGnRH-I nerve terminals in the median eminence suggests the presence of fine local regulation between these neuroendocrine systems.

Opiate pathways and neuropeptide Y (NPY) also contribute to cGnRH-I modulation during the ovulatory cycle. While naloxone administration stimulates cGnRH-I secretion, β-endorphin exerts an inhibitory effect, particularly near the preovulatory LH peak [280]. NPY gene expression increases under food restriction [281], correlating with decreased cGnRH-I expression [282]. In the median eminence, NPY promotes cGnRH-I release during the preovulatory LH surge, likely under progesterone influence, which reduces β-endorphin’s inhibitory effect [283].

Dopamine is another major regulator of the GnRH system, acting at both the hypothalamic and pituitary levels to stimulate LHβ subunit expression [284]. Dopaminergic neurons near cGnRH-I neurons are activated after photostimulation [285], and direct contacts between dopaminergic fibers and cGnRH-I neurons have been observed in sexually precocious birds or under food restriction [286,287].

Glial cells further modulate interactions between neurotransmitters, neuropeptides, and cGnRH-I neurons by shaping synaptic input, particularly in response to photostimulation. Studies in quail, chickens, and mammals indicate increased cFos expression, a marker of cellular activation, in the basal hypothalamus after light exposure, in both neurons and glial cells of the median eminence [288]. In quail, post-photostimulation cGnRH-I release is proposed to result from a local increase in T3 within the basal hypothalamus. Elevated T3 may induce glial retraction around GnRH-I terminals in the median eminence, facilitating hormone release [289].

The anterior pituitary contains gonadotropes, which respond to cGnRH-I released from the median eminence. These cells synthesize and secrete LH and FSH, each comprising a shared α-subunit and a hormone-specific β-subunit (LHβ or FSHβ). In chicks, LH and FSH are produced in distinct cell populations [290], providing an important basis for studying the separate regulatory mechanisms controlling each gonadotropin.

A critical component of reproductive regulation is the interaction between GnRH and its specific receptor (cGnRH-R) expressed on gonadotropes. Receptor binding activates intracellular signaling pathways, altering gene expression for gonadotropin subunits. Two GnRH isoforms (cGnRH-I and cGnRH-II) are present in the chicken hypothalamus [291]; both are capable of stimulating LH release in vitro, and in vivo studies have confirmed that either form can induce its secretion in chickens [292]. However, gonadotropin synthesis primarily relies on cGnRH-I receptor activation, as cGnRH-II is nearly absent from the median eminence [293].

Sex-specific differences exist in pituitary responses to cGnRH-I and cGnRH-II. In domestic chickens, LH release is greater in roosters than in hens. In roosters, both GnRH forms have similar potency, whereas in hens, cGnRH-II is more effective than cGnRH-I in stimulating LH release [294]. These differences likely arise from the differential regulation of cGnRH-RI and cGnRH-RII gene expression across the reproductive cycle, explaining the sexually dimorphic response to these peptides.

Unlike mammalian receptors, avian GnRH receptors have a cytoplasmic C-terminal tail that is involved in receptor internalization, ligand binding, phosphorylation, desensitization, and expression regulation [295]. In cGnRH-RII, this tail is longer than in cGnRH-RI, with conserved internalization regions [296]. Intracellular segments linked to transmembrane domains 3 and 7, crucial for G-protein coupling, are similar between both receptor types [297,298]. cGnRH-RI internalizes through a dynamin-dependent mechanism [299].

Receptor desensitization occurs with prolonged stimulation via receptor phosphorylation, which stabilizes β-arrestin binding, inhibits G-protein coupling, and inactivates downstream signaling [300,301].

In birds, GnRH receptors primarily couple with Gq proteins, activating phospholipase C (PLC) and triggering inositol phosphate signaling. This hydrolyzes phosphatidylinositol-4,5-bisphosphate (PIP2) into inositol-1,4,5-trisphosphate (IP3) and diacylglycerol (DAG). IP3 induces intracellular Ca^2+^ release, while DAG activates protein kinase C (PKC), initiating cascades that regulate gonadotropin subunit gene expression [302]. GnRH receptors may also interact with Gi and Gs proteins, affecting adenylate cyclase activity. Gs activation produces cAMP, which activates PKA, phosphorylating nuclear and cytoplasmic proteins, and modulating transcription through cAMP response elements. To date, both cGnRH-RI and cGnRH-RII signal mainly through Gq [303]. In addition, a third receptor subtype, GnRH-RIII, has been identified predominantly in the pituitary gland, where it shows a distinct expression and regulation pattern closely linked to reproductive activity and its functional interplay with the GnIH system [304].

Gonadotropin receptors are localized in the gonads, particularly in ovarian granulosa and theca cells and testicular Sertoli cells. In chickens, FSH receptor expression correlates with follicular responsiveness, peaking in the granulosa cells of small yellow follicles (6–8 mm), while theca cell expression remains relatively stable across follicular stages [305]. Preovulatory follicle hierarchy is established from small yellow follicles and involves reduced epidermal growth factor expression in selected follicles, which decreases its inhibitory effect on FSH-induced steroidogenesis in granulosa cells [306].

LH stimulates the expression of its own receptor but does not significantly affect FSH receptor expression. This is counterbalanced by activin A, particularly in mid-hierarchy preovulatory follicles undergoing rapid growth, which acts paracrinely to regulate LH sensitivity [307]. In prehierarchical follicles, activin A induces LH receptor expression and, together with FSH, promotes FSH receptor expression [308].

Long-term cell culture studies show LH receptor induction in granulosa cells exposed to both FSH and LH [309]. In vivo, LH receptor mRNA is expressed in granulosa cells of hierarchical follicles, increasing progressively with follicular maturation. In theca cells, LH receptor expression is present in prehierarchical follicles and increases up to the F2 follicle [310]. This enhanced LH receptor expression in granulosa cells reflects increased progesterone production, with the highest levels in mature preovulatory follicles.

### 8.3. The Effect of Light on the Hypothalamic-Pituitary-Gonadal (HPG) Axis

In controlled settings, the reproductive axis can be modulated by adjusting the photoperiod. Increasing the day length elevates both mRNA and GnRH-I peptide levels, which in turn activates pituitary gonadotropes and stimulates ovarian steroidogenesis [285,304], a response primarily mediated by deep-brain photoreceptors.

Conversely, the inhibitory pathway involving GnIH is regulated by melatonin [277,311], a hormone produced during the scotophase and predominantly present under short, non-stimulatory photoperiods (<12 h). Following photostimulation, rising levels of gonadal steroids (estradiol and progesterone) provide feedback to the pituitary, reducing GnIH receptor mRNA expression [312]. Additionally, longer day lengths lower melatonin secretion, which decreases GnIH levels and relieves its inhibitory effect on the stimulatory reproductive pathway [313].

Numerous studies have reported the influence of light on hormone levels in poultry. For instance, Han et al. [314] found that extending the photoperiod enhances FSH and LH secretion in pre-laying hens. Similarly, in 20-week-old Cobb breeder chicks, Lewis et al. [315] observed that longer photoperiods were associated with increased LH levels, with the most pronounced rise at 11 h of light and a plateau at 13 h. The findings of Mengqian Liu et al. [316] are generally consistent with this, although variations may arise due to factors such as age at light stimulation initiation, differences in lighting schedules, or the chicken breeds examined.

In line with this, the authors of [316] investigated the effects of varying photoperiods on reproductive hormone levels in the hypothalamus and pituitary of male Chahua Chickens No. 2. A transcriptomic analysis was also conducted to clarify the molecular mechanisms through which the photoperiod regulates hormone secretion. The findings indicated that extending the photoperiod led to significant increases in blood levels of GnRH, FSH, and LH, confirming its strong stimulatory effect on reproductive hormone production.

Beyond photoperiod, light quality-specifically the number of photons that reach the bird and the emission spectrum-also plays a key role. Longer wavelengths penetrate the skull and brain tissue more effectively, stimulating hypothalamic photoreceptors [317]. As a result, red light is the most effective in activating the reproductive axis [41,318]. Shorter wavelengths, such as blue or green light, require higher intensities to elicit the same hypothalamic response [319], and evidence suggests that green light may even inhibit reproduction via retinal photoreceptors [320]. While the impact of green light on growth in laying hens is less clear, studies in both hens and roosters have shown that green light exposure can enhance body weight gain [41,321].

Overall, these findings highlight that both photoperiod and light characteristics critically influence the avian reproductive axis. Longer day lengths and light of longer wavelengths, particularly red light, stimulate hypothalamic photoreceptors, enhance GnRH-I activity, and promote gonadal steroidogenesis. In contrast, shorter photoperiods and inhibitory signals mediated by melatonin and GnIH suppress reproductive activity. Understanding these photic and hormonal interactions provides valuable insights for optimizing reproductive performance and growth in poultry under controlled environmental conditions.

## 9. Hypothalamic-Pituitary-Thyroid Axis

### 9.1. Anatomy and Histology of the Thyroid Gland

The avian thyroid gland is an oval-shaped, highly vascularized endocrine organ. Unlike mammals, birds possess two distinct lobes-left and right-located on either side of the trachea, near the posterior larynx, at the junction where the carotid arteries meet the subclavian arteries [322]. Histologically, the thyroid structure in birds shows no major differences compared with other vertebrates. The gland is predominantly composed of thyroid follicles, which make up around 80% of its mass, consisting of a single layer of epithelial cells called thyrocytes, which have a three-dimensional oval shape. When stimulated by thyroid-stimulating hormone (TSH), these cells become columnar; in a resting state, they appear flattened. The center of each follicle is filled with a protein-rich colloid, surrounded by the epithelial layer. The main component of this colloid is thyroglobulin (TG), a glycoprotein with two major roles: (1) it acts as a precursor for thyroid hormone (TH) synthesis due to the presence of tyrosyl residues and (2) serves as a large intrathyroidal T3 and T4 reservoir. This hormone storage mechanism is a unique adaptation that helps the organism cope with periodic iodine deficiency. The outer boundary of the follicle is formed by the basal membrane of the thyrocytes, which is in direct contact with an extensive capillary network, facilitating efficient exchange between the blood and follicular lumen. Tight junctions between these cells create a selective barrier, preventing the unwanted diffusion of transmembrane proteins and uncontrolled hormone release into circulation. In addition, parafollicular or C cells are found between follicles. These cells are involved in the production and secretion of calcitonin, a hormone that plays a role in calcium and phosphate metabolism [323].

### 9.2. Hormonal Influence on Tissues

**Figure 4:**
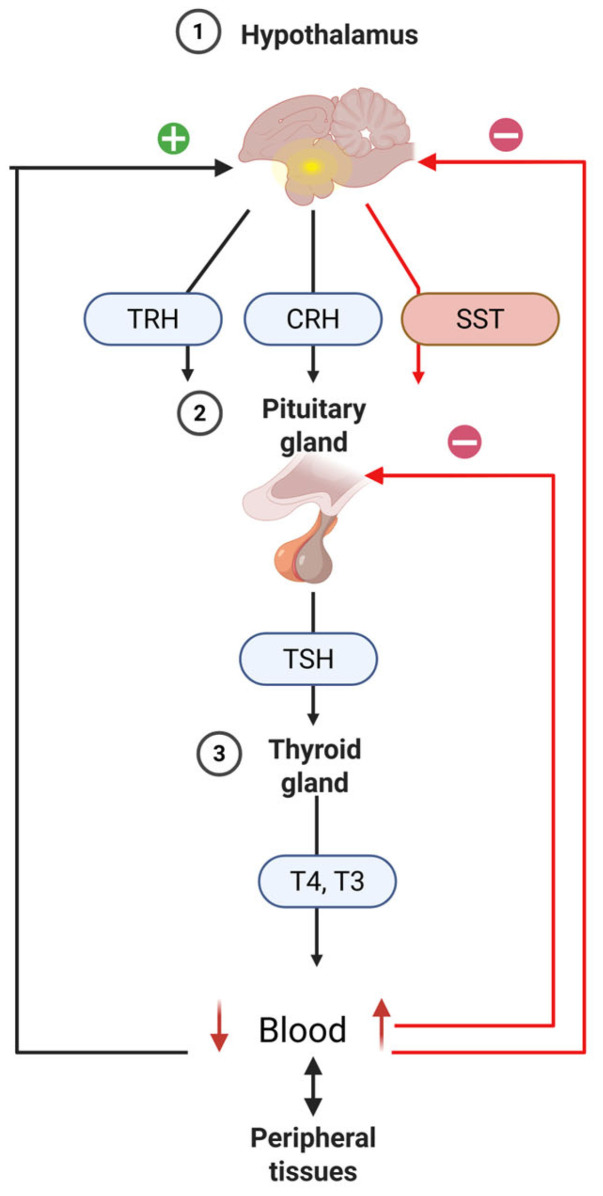
The hypothalamic-pituitary-thyroid axis. TRH-thyrotropin-releasing hormone; CRH-corticotropin; SST-somatostatin; TSH-thyroid-stimulating hormone; T4-thyroxine; T3-triiodothyronine. Created in BioRender. https://BioRender.com/wvfhszc (accessed on 20 October 2025).

The hypothalamic-pituitary-thyroid (HPT) axis is an endocrine system regulated by a negative feedback loop. When the body experiences a deficiency in thyroid hormones (THs), the hypothalamus-specifically the paraventricular nucleus (PVN)-releases thyrotropin releasing hormone (TRH). This hormone travels through the hypothalamo-hypophyseal portal circulation to the pituitary gland where it promotes thyroid stimulating hormone (TSH) production and release into the bloodstream. TSH binds to its specific receptor on thyroid cells, triggering thyroid hormone production and secretion [151]. During embryonic development and growth, TRH influences pituitary cell function [324,325]. In adult birds, however, its effect on thyroid function becomes less pronounced, while its role in stimulating GH secretion increases [325]. TSH encourages thyroid gland enhancement and boosts the synthesis and release of T4 and, to a lesser extent, T3. These hormones circulate in the blood both freely and bound to transport proteins. Once the concentration of T4 and T3 in the blood reaches appropriate levels, they exert negative feedback on the hypothalamus and pituitary, reducing the secretion of TRH and TSH (Figure 4). In birds, corticotropin-releasing hormone (CRH) also acts as a significant thyrotropic factor, working via CRHR2 receptors to stimulate TSH synthesis and release from anterior pituitary thyrotropes [326]. Besides TRH and CRH, the HPT axis is further regulated by somatostatin (SST), a hypothalamic peptide that suppresses TSH production in the pituitary [327]. TSH activates thyroid cells by binding to its receptor and promotes TH production and release [151].

Thyroid hormones (THs), namely, thyroxine (T4) and triiodothyronine (T3), are derived from thyroglobulin and secreted by the thyroid gland [328]. These hormones are critical basal metabolic rate and systemic homeostasis regulators. They are indispensable for normal development and growth and contribute significantly to the proper function of the nervous, muscular, circulatory, and reproductive systems. In birds, T4 is the primary hormone synthesized by the thyroid gland, while T3 is mainly produced outside the thyroid through the enzymatic conversion (deiodination) of T4. Evidence shows that under both resting conditions and during hormonal stimulation, the avian thyroid predominantly secretes T4, which accounts for over 99% of total TH output, whereas T3 is released in only minimal amounts [329].

T4 is considered less potent than T3 due to its reduced thyroid hormone receptor binding affinity [152]. In circulation, both hormones are bound to carrier proteins such as transthyretin (TTR), albumin, and thyroxine-binding globulin (TBG) [330]. These transport proteins enhance solubility in plasma and facilitate hormone delivery to target tissues by preventing their passive diffusion across lipid membranes. Once inside the target tissue, T4 is locally converted to T3 by deiodinase enzymes DIO1 and DIO2, which remove an iodine atom from its outer ring [154,155]. The liver is also a major site of T3 production, representing its primary source [331]. T3 has a much higher binding affinity for receptors compared with T4. It acts primarily through nuclear thyroid hormone receptors, THRA and THRB, which activate either after direct cellular uptake or following intracellular conversion from T4.

To maintain appropriate hormonal signaling, T3 can be deactivated locally in target tissues by DIO1 or DIO3 through its conversion into 3,5-diiodo-L-thyronine (T2). Additionally, DIO3 may block the formation of T3 from T4 by converting the latter into reverse T3 (rT3) [332,333]. Deiodinase enzymes provide a mechanism for tissue-specific thyroid hormone action regulation. Thus, plasma THs levels do not always accurately reflect the TH activity at the tissue level due to the localized nature of these enzymes. Type I deiodinase (DIO1) is predominantly found in the liver, kidneys, and muscle, while Type II deiodinase (DIO2) is primarily expressed in the brain [334], particularly in the pituitary, where it is responsible for producing over 75% of the local T3. In contrast, Type III deiodinase (DIO3) is widely distributed in many tissues. During embryonic development, its highest activity is seen in the liver and kidneys, and in adult birds, it is also present in the brain, pituitary, heart, skeletal muscle, thyroid, and skin [322]. Thyroid hormone receptors (THRs) are located in the cell nucleus and act as T3 binding-activated transcription factors [335].

Free thyroid hormones (THs) travel through the bloodstream to reach their target tissues. To regulate gene expression, they must cross the cell membrane. Research has shown that specific transmembrane proteins facilitate iodothyronine transport into and out of cells [336]. These transporters belong to three families: (i) monocarboxylate transporters (MCTs), (ii) organic anion transporting polypeptides (OATPs), and (iii) L-type amino acid transporters (LATs). These systems primarily transport T4, which is then converted to T3 inside the cell. All THRs contain a nuclear localization signal [337], which ensures their proper transport into the nucleus, often with the help of N-terminal peptides [338]. Once T3 binds to THRs at thyroid hormone response elements on DNA, conformational changes allow co-regulator and transcriptional machinery recruitment, modulating gene expression [339].

### 9.3. Hormone-Mediated Regulatory Signaling

The thyrotropic axis is centrally regulated by neurons in the paraventricular nucleus (PVN) that secrete thyrotropin-releasing hormone (TRH). These neurons play a critical role in maintaining the normal activity of the axis. TRH expression is influenced by various hormones, most notably from the thyroid, which exert negative feedback control: elevated TH levels suppress TRH production, while low levels enhance it [340]. Other key modulators include leptin [341], α-melanocyte-stimulating hormone (α-MSH), and noradrenaline (NA) [342]. Once produced in the hypothalamus, TRH enters the portal circulation and stimulates TSH secretion from the anterior pituitary thyrotrophs by binding to TRH receptor 1 (TRHR-1), a GPCR [343]. In chickens, both TRHR-1 and TRHR-3 have been identified [344]. TRHR-1 activation triggers signaling cascades via cAMP or IP3, leading to the recruitment of transcription factors such as CREB, AP-1, and Elk-1 [146,147].

TSH primarily acts on the thyroid gland, promoting TH synthesis. Its receptor, TSHR, is located on the basolateral membrane of thyroid follicular cells [345] and is also a GPCR. When activated, thyroglobulin is mobilized from the colloid and internalized into follicular cells [328]. This glycoprotein serves as both a scaffold and a precursor for thyroid hormone synthesis [346], and is present in chickens both before and after hatching [347,348]. TSH enhances thyroglobulin gene expression [349]. Within the follicular cells, tyrosine residues on thyroglobulin undergo iodination, forming monoiodotyrosine (MIT) and diiodotyrosine (DIT), which subsequently couple to produce THs. T4 results from two DITs, while T3 is formed from a DIT and an MIT [350,351]. Although thyroid hormone release was once believed to occur through passive diffusion, studies show that low plasma TH levels occur when transport proteins like MCT8 are dysfunctional [352]. This suggests that a dual mechanism involving both passive and active transport is required to maintain TH homeostasis.

Thyroid hormone signaling involves multiple mechanisms. THRs mediate genomic responses by binding T3 intracellularly and regulating gene expression through interaction with thyroid hormone response elements (TREs) [353,354]. Ligand binding induces structural changes that enhance receptor affinity for TREs [355]. These elements may contain palindromic half-sites, and THRs can form monomers, homodimers, or heterodimers (THRA/THRB) to modulate transcription differently [356,357,358]. Their action is further strengthened by coactivator proteins (TRAPs) [359].

A second essential component of TH regulation is the deiodinase (DIO) enzyme system, which, although not directly involved in receptor signaling, modulates hormone bioavailability by removing iodine atoms from THs, thereby fine-tuning their activity in a tissue-specific manner [360].

In conclusion, the proper functioning of the thyrotropic axis depends on an integrated network of regulatory mechanisms, including the release of TRH and TSH, the synthesis and iodination of thyroglobulin for thyroid hormone production, their transport and secretion into the circulation, and deiodinase (DIO) enzymatic activity. Collectively, these processes establish a highly coordinated and dynamic regulatory system that is essential for development, growth, and metabolic homeostasis maintenance in both birds and mammals.

### 9.4. The Effect of Light on the Hypothalamic-Pituitary-Thyroid (HPT) Axis

Thyroid hormones are characterized by distinct circadian and/or seasonal secretion patterns and are highly sensitive to photoperiodic changes. As such, exposure to artificial light at night (ALAN) may significantly affect their secretion, thereby influencing a wide range of physiological processes. This section provides a brief overview of THs’ involvement in regulating circadian and seasonal rhythms, outlining the potential effects of ALAN on thyroid function.

T3 and T4 play critical roles in growth regulation [361]. Their levels are positively associated with feed intake in chicks [362,363], and they have been implicated in both growth suppression and compensatory growth stimulation in broilers [364]. Beyond their role in growth, THs are essential regulators of basal metabolic rate and are actively involved in thermoregulatory processes. They are indispensable for the proper development and function of nearly all tissues. Moreover, they contribute to photoperiodic regulation, modulate the hypothalamic-pituitary-gonadal axis, and play a part in the molting cycle.

At the cellular level, the physiological effects of THs are mediated by specific nuclear and/or membrane receptors and depend on hormone availability. In birds, since the thyroid gland produces only limited amounts of T3, its circulating levels are largely determined by deiodinase expression and activity, enzymes responsible for the local conversion and metabolism of this iodothyronine in peripheral tissues. Seasonal variation in the timing of peak T3 and T4 levels suggests a strong photoperiodic influence on thyroid activity [365]. This variation highlights the fundamental role of thyroid hormones in the photoperiodic responses of vertebrates, acting as central mediators of physiological adaptation to changes in day length [366,367]. ALAN can interfere with the organism’s ability to accurately perceive natural day length, leading the neuroendocrine system to interpret such lighting conditions as an extension of the photoperiod and mimicking a long-day environment. As a result, ALAN exposure may profoundly reshape endocrine and physiological states, promoting a shift toward long-day phenotypes. Within the context of thyroid function, extended photoperiods initiate a physiological cascade that enhances the peripheral conversion of T4 to the more biologically active T3, a process that plays a key role in gonadal activation. Elevated TSH and T3 levels under long-day conditions have been documented across several species, further supporting the involvement of this mechanism [368].

In addition to photoperiod duration, the spectral composition of light can modulate thyroid hormone dynamics in birds. This is exemplified by studies reporting significantly higher plasma T4 concentrations in birds exposed to GL compared with those maintained under WL, despite identical photoperiodic conditions [369].

Shahabodin Gharahveysi [13] reported that broiler chicks reared under green light achieved greater body weight compared with those exposed to yellow or red. This outcome was linked to modifications in thyroid hormone and testosterone secretion, both of which are known to directly influence metabolic activity and muscle development.

Green light is thought to reduce metabolic rate by regulating thyroid hormone levels, thereby improving energy efficiency and facilitating increased body mass accumulation [370]. Moreover, this specific light spectrum has been shown to enhance muscle growth, particularly during the early phases of development, as evidenced by several previous studies [7,197,262]. Nonetheless, it is important to note that changes in circulating levels of thyroid hormones and other blood metabolites were not consistently significant across all experimental conditions. Rozenboim [194] explored the effects of age-dependent combinations of green and blue light on growth performance and T3 concentrations, though no clear and consistent relationship was established. Although the highest T3 concentration was found in the heaviest chicks from a group exposed to a transition from blue to green LED lighting at 20 days of age, this correlation was not uniformly observed. In contrast, environmental temperature appeared to have a more pronounced effect on metabolic function than lighting conditions, with a significant correlation reported between ambient temperature and T3 levels, as demonstrated in earlier research [364,371]. Liu et al. [370] provided evidence that various combinations of monochromatic light can substantially influence the circulating thyroid hormone levels in broiler chickens. More specifically, exposure to green-blue light was associated with significantly lower concentrations of T4, FT4, FT3, and TSH, while the red–red light combination led to the highest hormonal levels.

The variations observed were accompanied by shifts in gene expression related to muscle fiber types. A negative association was found between FT3 levels and genes linked to oxidative fibers, whereas a positive association emerged with those associated with glycolytic fibers. These patterns indicate that thyroid hormones may directly contribute to muscle fiber-type transitions triggered by light stimuli. This aligns with the broader understanding that broiler muscle phenotype development is governed by intricate interactions between genetic determinants and external modulators such as neural signaling, contractile activity, and hormonal milieu. Among the endocrine regulators involved, T3 plays a central role by acting through its specific nuclear receptors, influencing the transcription of genes coding for different myosin heavy chain (MHC) isoforms [372]. Reduced TH availability tends to favor slow-twitch fiber trait expression, while elevated levels promote fast-twitch, glycolytic fiber development. This regulatory mechanism has been further substantiated by thyroidectomy experiments, which demonstrated that in the absence of THs, the modulatory effects of light on muscle fiber-type differentiation are eliminated.

The enzymes DIO2 and DIO3 play essential roles in intracellular TH activity regulation, functioning as key modulators of hormonal activation and inactivation pathways [373]. DIO2 facilitates prohormone T4 conversion into its active form, T3, thereby enhancing its local bioavailability and intensifying its physiological effects. Conversely, DIO3 acts as an inactivating enzyme by converting T4 into reverse T3 (rT3) and T3 into T2, effectively dampening T3′s classical nuclear actions [374]. Sagliocchi et al. [375] further linked DIO2 upregulation to metabolic adjustments favoring the expression of faster and more glycolytic MHC isoforms, suggesting that these deiodinases play a direct role in shaping muscle fiber characteristics. Light environment appears to influence these enzymatic pathways. Exposure to green-blue light was associated with increased DIO3 expression and decreased DIO2 expression, while the opposite pattern was observed under red–red lighting conditions. These molecular alterations mirrored the observed differences in serum thyroid hormone concentrations, indicating that specific light spectra may modulate intramuscular T3 levels through differential deiodinase activity regulation. This mechanism likely contributes to the observed shifts in muscle fiber phenotype under varying light conditions.

TH levels are also tightly linked to energy metabolism and body mass regulation [376]. Elevated TH levels, such as those seen in hyperthyroid states, increase basal metabolic rate and resting energy expenditure, often leading to weight loss despite higher food intake. This paradox arises from enhanced futile metabolic cycling and reduced energy utilization efficiency [155]. Beyond their metabolic role, thyroid hormones are involved in regulating oxidative stress by influencing reactive oxygen species production in target tissues [377]. Interestingly, slightly lower TH levels have been associated with protective effects, potentially due to reduced oxidative stress and improved cellular resilience [378]. The results emphasize that the influence of light on broiler development is closely linked to interactions with other environmental factors, such as temperature, and largely mediated by the thyroid axis. Through the TH-DIO2/DIO3 signaling pathway, thyroid hormones play a central role in regulating muscle fiber type conversion and growth efficiency. In particular, illumination with blue-green light modulates this hormonal pathway, favoring oxidative fiber development, muscle hypertrophy and antioxidant capacity. These findings support lighting regime optimization as a viable strategy for improving meat quality and productive performance in intensive rearing systems.

## 10. Hypothalamus-Pituitary-Adrenal Axis

### 10.1. Anatomy and Histology of the Adrenal Glands

Present in pairs, the adrenal glands are positioned anterior and medial to the cranial pole of the kidney. Despite considerable variation in their shape across species, they are typically flattened and lie in close proximity, occasionally fusing in some animals. These glands receive arterial blood primarily from the cranial renal arteries, and sometimes directly from the aorta. Each adrenal gland drains through a single adrenal vein into the caudal (posterior) vena cava. Additionally, in chicks, one or two lymphatic vessels are associated with each gland [379]. Preganglionic sympathetic fibers from the thoracic and sacral splanchnic nerves [380] converge toward cranial and caudal ganglia, which are either located on the pericapsular sheath or integrated within the glandular tissue. These predominantly cholinergic preganglionic fibers bypass the ganglia and directly innervate chromaffin cell clusters within the gland. Each cluster typically contains 12 or more chromaffin cells [381]. In the adrenal gland, the subcapsular area consists exclusively of medullary tissue.

Originating from the ganglia, postganglionic sympathetic fibers, which are noradrenergic in nature, supply the adrenal blood vessels and may also target chromaffin cells [382]. These fibers also contribute to the formation of renal and gonadal nerve plexuses. Some evidence indicates that, as in mammals, the avian adrenal gland may also receive parasympathetic innervation, likely mediated by the vagus nerve through the celiac plexus [380]. Notably, adrenal blood vessels are innervated by both cholinergic and adrenergic terminals [382]. While synaptic endings are located close to adrenocortical cells, direct synaptic contacts are infrequent [383].

In chicks, the adrenal gland is surrounded by a dense fibrous connective tissue capsule, which is rich in collagen, reticular fibers, and blood vessels, but contains few elastic fibers [384,385]. The capsule’s thickness shows sexual dimorphism, as is significantly greater in females (22.09 ± 2.17 µm) than in males (18.14 ± 1.82 µm) [385]. Trabeculae extend from the capsule into the glandular parenchyma, contributing to its internal structure [384].

The avian adrenal parenchyma is composed of a combination of adrenocortical and chromaffin tissues. Chromaffin cells are predominantly concentrated in the central region of the gland, an area characterized by rich vascularization. Similar to in mammals, chromaffin cells are organized into small clusters of approximately 10–12. These cells are basophilic, polygonal or round, with basophilic cytoplasm and a large spherical nucleus containing two to three nucleoli [386]. Each cluster is typically innervated by a single nerve fiber bundle. However, in birds, these clusters do not merge to form the distinct adrenal medulla seen in mammals. A single nerve terminal may establish synaptic contact with up to three chromaffin cells of the same type-either NA or adrenaline (AD) secreting [381].

In broiler chicks, male adrenal cells exhibit slightly greater cell height (8.72 ± 0.231 µm) and nuclear diameter (4.39 ± 0.359 µm) compared with those from females (8.67 ± 0.218 and 3.84 ± 0.326 µm, respectively) [387]. The cytoplasm of these cells contains mitochondria with tubular cristae, ribosomes, endoplasmic reticulum, lipid droplets, and secretory granules [384,388]. Ganglionic cells are also observed interspersed between the medullary chromaffin cells [384]. In chick adrenal glands, approximately 70% of the chromaffin cells are AD type [389]. A consistent pattern has been observed in the spatial distribution of these cells: NA chromaffin cells are typically found at the edges of clusters, while AD cells are generally located toward the center [390,391].

Adrenocortical cells are organized into cords that extend outward from the gland’s center, forming frequent branches and connections. At the periphery, these cords loop along the inner side of the connective tissue capsule. Each cord comprises two rows of adrenocortical cells aligned with their long axes positioned perpendicular to the cord’s direction. Typically, though with some variations, the nuclei of these cells are eccentrically positioned near the cord’s shared, non-sinusoidal basal lamina [304]. The cellular heterogeneity-seen both microscopically and ultrastructurally-suggests the presence of a primitive form of zonation. Peripheral adrenocortical cells tend to be larger, often binucleated, and rich in lipid droplets, whereas those closer to the gland’s center are usually smaller, more elongated, and contain fewer lipids [392]. In birds, the structural organization of cortical (interrenal) and medullary (chromaffin) cell strands defines the zonation pattern within the adrenal parenchyma [323]. In hens, distinct peripheral (subcapsular) and central (internal) zones are observable upon adrenal gland sectioning [384]. Humayun et al. [393] also identified a subcapsular layer, as well as peripheral and central zones in the chick adrenal gland, consistent with similar observations in partridges [394] and quails [395].

### 10.2. Hormonal Influence on Tissues

The adrenocorticotropic axis is composed of CRH, ACTH, and CORT. CRH is synthesized by the PVN of the hypothalamus in response to environmental stressors [396], such as ambient temperature in broiler housing, stocking density, and limited access to water or feed. CRH release stimulates the anterior pituitary to secrete ACTH [138,141], which then promotes CORT production by the adrenal cortex [142]. This regulatory pathway is highly conserved across both mammals and avian species [397,398]. As the primary glucocorticoid in birds, CORT plays a fundamental role in triggering cellular mechanisms that mediate the stress response and modulating metabolic activity in reaction to stressors. Glucocorticoids are a diverse group of steroid hormones produced from cholesterol within the adrenal cortex [399]. While cortisol serves as the main circulating glucocorticoid in most mammals (with the exception of rodents), CORT fulfills this role in birds [400]. Due to its lipophilic nature, CORT does not circulate freely in the bloodstream; instead, it binds to corticosteroid-binding globulin (CBG), also known as transcortin [401], which is primarily synthesized in the liver [402]. In humans, CBG transports cortisol by enclosing it within a hydrophobic binding pocket [403,404], enabling its delivery to various target organs such as muscles, bones, liver, intestines, and kidneys [405,406]. Despite only a fifty percent similarity in amino acid composition at the steroid-binding site, avian CBG performs a comparable function to its mammalian counterpart [407].

### 10.3. Hormone-Mediated Regulatory Signaling

In the presence of an external stressor [137], the PVN of the hypothalamus releases CRH [139], which binds to specific CRH receptors (CRHRs). In chickens, two receptor subtypes have been identified: CRHR1 and CRHR2 [140,144]. Both are G protein-coupled receptors (GPCRs) that transmit CRH signals, although the latter has a notably higher binding affinity for CRH than the former [140]. Similar to GHRH receptors, CRHRs initiate intracellular signaling cascades, predominantly the cAMP/PKA pathway, which activates ACTH synthesis through the stepwise cleavage of the precursor proopiomelanocortin (POMC) by prohormone convertase 1/3 (PC1/3) [408,409,410]. It is also suggested that CRHRs may promote ACTH production by forming dimers with the vasotocin receptor subtype VT2R [411].

Following ACTH release from the anterior pituitary, it fulfills two primary roles: stimulating CORT secretion from the adrenal cortex and inhibiting further CRH release from the hypothalamus [412,413]. Similar to CRH, ACTH exerts its effects through a GPCR known as the melanocortin-2 receptor (MC2R) [414]. Although MC2R signaling mechanisms in chickens remain understudied, its structural similarity to mammalian MC2R implies a comparable function [415]. For functional activity, MC2R requires the presence of melanocortin receptor accessory proteins (MRAPs) [416], of which three essential ones have been identified in zebrafish and one in chickens [417,418]. These proteins facilitate MC2R transport from the endoplasmic reticulum to the plasma membrane, enabling ACTH signaling [419,420].

Corticosterone exerts its effects through the glucocorticoid receptor NR3C1, a ligand-dependent transcription factor. Upon reaching its target tissue, CORT diffuses into the cell and binds NR3C1 in the cytoplasm. At rest, NR3C1 is part of a chaperone complex including essential proteins HSP90 and HSP70, along with auxiliary components such as Hop, HSP40, and p23 [421]. Receptor inactivation is initiated by HSP70-induced partial unfolding, while reactivation depends on ATP hydrolysis by HSP90, a process regulated by histone deacetylase 6 (HDAC6) [422,423]. Upon ligand binding, NR3C1 dissociates from the chaperone complex and translocates into the nucleus, where it binds to glucocorticoid response elements (GREs) in regulatory regions of specific genes, leading to either transcriptional activation or repression depending on the tissue context [424,425]. In addition to its cellular effects, CORT contributes to a classical negative feedback loop by suppressing CRH and ACTH secretion at hypothalamus and anterior pituitary levels, respectively [426].

### 10.4. The Effect of Light on the Hypothalamic–Pituitary–Adrenal (HPA) Axis

Both within individual cells and across organ systems, circadian rhythms play a fundamental role in regulating numerous physiological activities by ensuring synchronized functioning between various biological systems. The adrenergic system is one such example, relying on the catecholamine neurotransmitters-adrenaline and noradrenaline-to perform essential biological tasks. The synthesis and release of these catecholamines are governed by both neural inputs from the sympathetic nervous system and hormonal signals involving the HPA axis. Notably, both regulatory pathways are influenced by signals from the central circadian clock, which contributes to the observed rhythmic fluctuations in catecholamine concentrations in the bloodstream and peripheral tissues. These oscillations can affect a range of target cells expressing adrenergic receptors, including those within the immune system [427].

The HPA axis is a central regulator of the body’s response to environmental stressors and external challenges [428]. Glucocorticoid hormones produced by the adrenal glands are essential for maintaining energy homeostasis in vertebrates [429]. These hormones enable the organism to respond to both routine environmental variations and sudden, acute stressors. As such, glucocorticoids serve as key modulators of phenotypic plasticity, allowing individuals to adapt to changing ecological conditions [430].

ALAN represents a relatively recent environmental stressor, the effects of which on HPA axis functioning are not yet fully understood. In particular, the extent to which ALAN influences the expression, localization, and sensitivity of glucocorticoid receptors (GRs) remains unclear. A thorough understanding of ALAN’s impact on the HPA axis requires an examination of both axis reactivity-meaning its activation and the resulting increase in circulating glucocorticoids (GCs) under stress-and the efficiency of its negative feedback regulation, through which GCs inhibit further hormone secretion by acting on GRs located in central structures such as the hypothalamus and anterior pituitary. It is possible that ALAN disrupts this feedback mechanism or alters GR expression or functionality, potentially leading to impaired glucocorticoid regulation and persistent HPA axis activation. Such dysregulation may have negative physiological and behavioral consequences. Therefore, only an integrated investigation of the entire HPA axis-from stress perception to hormone production and receptor-mediated feedback control-will allow for a comprehensive understanding of the mechanisms through which ALAN interferes with endocrine balance and for the development of effective mitigation strategies [24].

In chickens, as in other vertebrate species, the HPA axis functions as the primary system responsible for mediating long-term responses to stress. Upon activation, it stimulates the pituitary gland to secrete ACTH. However, inconsistent findings regarding the influence of light color on the physiological stress response have been reported in the scientific literature [259]. Prayitno et al. [5] and Hesham et al. [431] reported that broiler chickens exposed to red light exhibited increased aggressive behavior compared with those housed under blue, green, or white light. Additionally, Archer [432] demonstrated that exposing eggs to light, particularly white and red, during incubation subsequently reduced fear responses in chicks compared with incubation in darkness or under green light. Olanrewaju et al. [433,434] found no significant differences in corticosterone levels between birds raised under different LED light colors. Xie et al. [435] proposed that blue light might help mitigate stress responses in broilers, potentially through reducing serum interleukin-1β levels. Similarly, the findings of Helva [259] and Franco et al. [436] suggest that blue and green light may alleviate the negative effects of stress, supporting the conclusions drawn by Xie et al. [435]. Conversely, Mehaisen et al. [437] noted that glucocorticoid hormones suppress growth and feed intake. The elevated ACTH levels observed under white light conditions may be an additional factor contributing to reduced feed consumption in broilers exposed to this type of lighting.

## 11. Conclusions

Hypothalamic-pituitary-somatotropic (HPS) axis: Exposure to green LED light stimulates hypothalamic GHRH expression, pituitary GH secretion, and hepatic and muscular IGF-1 production, activating the JAK2/STAT5 and mTORC1 pathways that promote satellite cell proliferation and early muscle hypertrophy. Furthermore, melatonin receptors Mel1b and Mel1c participate in mediating the growth hormone secretion induced by green light in chicken adenohypophysis cells through the AC/PKA and ERK1/2 signaling pathways. In contrast, red light is associated with increased hypothalamic somatostatin expression and reduced pulsatile GH secretion, thereby limiting early muscle growth stimulation.

Hypothalamic-pituitary-gonadal (HPG) axis: The hypothalamus regulates reproductive function in birds by integrating hormonal and photic signals, maintaining the GnRH and GnIH balance that controls gonadotropin secretion. The activity of these neurohormones is influenced by melatonin, steroid and thyroid hormones, and environmental cues, ensuring reproductive cycle synchronization with seasonal conditions. Photoperiod and light quality play a crucial role: long days and red light stimulate hypothalamic and gonadal activity, while short days and melatonin inhibit reproduction. The interaction between endocrine and light-derived signals thus ensures precise hypothalamic-pituitary-gonadal axis regulation in birds.

Hypothalamic-pituitary-thyroid (HPT) axis: Variations in photoperiod and ALAN adjust TRH-TSH-T_4_/T_3_ cascades and deiodinase-mediated local T_3_ availability, modulating basal metabolism, seasonal reproductive cues, and tissue-specific thyroid hormone action through THRA/THRB signaling.

Hypothalamic-pituitary-adrenal (HPA) axis: Environmental stressors and the light spectrum interact in the CRH-ACTH-CORT feedback system: red light amplifies HPA activation and stress behaviors, whereas blue/green illumination mitigates corticosterone release and inflammation, indicating that spectral tuning can buffer stress responses.

In conclusion, exposure to green and blue light exerts a markedly superior enhancement of poultry productivity compared with red and white light. Furthermore, the controlled manipulation of LED spectral quality and exposure regimes provides a precise means of optimizing broiler growth, metabolic homeostasis, and stress resilience, thereby supporting sustainable and welfare-oriented poultry production practices.

## Data Availability

The original contributions presented in this study are included in the article. Further inquiries can be directed to the corresponding author.

## Abbreviations

The following abbreviations are used in this manuscript:

LED	Light-emitting diode
GHRH	Growth hormone-releasing hormone
IGF-1	Insulin-like growth factor 1
GnRH	gonadotropin-releasing hormone
LH	luteinizing hormone
FSH	follicle-stimulating hormone
TRH	Thyrotropin-releasing hormone
TSH	Thyroid-stimulating hormone
T4	Thyroxine
T3	Triiodothyronine
CRH	Corticotropin-releasing hormone
ACTH	Adrenocorticotropic hormone
ALAN	Artificial light at night
DBPs	Deep Brain Photoreceptors
SCN	Suprachiasmatic nucleus
VA opsin	Vertebrate Ancient opsin
MBH	Mediobasal hypothalamus
Per1	Period Circadian Regulator 1
cAMP	Cyclic adenosine monophosphate
HPA	Hpothalamic–pituitary–adrenal
SCG	Superior cervical ganglia
Pit-1	Pituitary-specific transcription factor 1
Mel 1b	Melatonin receptor type 1b
Mel 1c	Melatonin receptor type 1c
SST	Somatostatin
GHRL	Ghrelin
Mel 1a	Melatonin receptor type 1a
POA	Preoptic area
GHR	Growth hormone receptor
IGFs	Insulin-like growing factors
IGFR1	Insulin-like Growth Factor 1 Receptor
IGFBPs	Insulin-like growth factor binding proteins
CORT	Corticosterone
NR3C1	Nuclear Receptor Subfamily 3, Group C, Member 1
CBG	Corticosteroid-Binding Globulin
FCR	Feed conversion ratio
DIOs	Deiodinases
ECM	Extracellular matrix
HGF	Hepatocyte growth factor
FGF2	Fibroblast growth factor 2
IGF	Insulin-like growth factor
TGF-β	Transforming growth factor-beta
MyoD1	Myoblast determination protein 1
MYF5	Myogenic factor 5
MRFs	Myogenic regulatory factors
IGF2	Insulin-like Growth Factor 2
THs	Thyroid hormones
SSTR2	Somatostatin receptor 2
IGFBP1	Insulin-like Growth Factor Binding Protein 1
IGFBP7	Insulin-like Growth Factor Binding Protein 7
IGFBP6	Insulin-like Growth Factor Binding Protein 6
IGFALS	Insulin-like Growth Factor Acid-Labile Subunit
IGFBP3	Insulin-like Growth Factor Binding Protein 3
IGFBP2	Insulin-like Growth Factor Binding Protein 2
IGFBP4	Insulin-like Growth Factor Binding Protein 4
IGFBP5	Insulin-like Growth Factor Binding Protein 5
PVN	Hypothalamic paraventricular nucleus
GPCR	G Protein-Coupled Receptor
ATP	Adenosine Triphosphate
PKA	Protein kinase A
CREB	cAMP Response Element-Binding protein
SSTR2A	Somatostatin Receptor 2A
SSTR2B	Somatostatin Receptor 2B
RL	Red light
GL	Green light
SSTR1–5	Somatostatin Receptors 1 to 5
JAK2/STAT5	Janus Kinase 2/Signal Transducer and Activator of Transcription 5
Src/ERK	Src kinase/Extracellular signal-Regulated Kinase
Akt	Protein Kinase B
mTOR	Mechanistic Target of Rapamycin
TSC2	Tuberous sclerosis complex 2
RPTOR	Regulatory Associated Protein of mTOR
mTORC1	Mechanistic Target of Rapamycin Complex 1
SREBPs	Sterol regulatory element-binding proteins
S6Ks	Ribosomal Protein S6 kinases
BL	Blue light
PCNA	Proliferating Cell Nuclear Antigen
WL	White light
BDNF	Brain-Derived Neurotrophic Factor
TrkB	Ropomyosin receptor kinase B
ERK	Extracellular signal-regulated kinase
GnIH	Gonadotropin-Inhibitory Hormone
cGnRH-I	chicken Gonadotropin-Releasing Hormone I
HPG	hypothalamo–pituitary–gonadal axis
VIP	Vasoactive intestinal peptide
NPY	neuropeptide Y
cGnRH-R	chicken Gonadotropin-Releasing Hormone Receptor
cGnRH-II	chicken Gonadotropin-Releasing Hormone II
PLC	phospholipase C
PIP2	phosphatidylinositol-4,5-bisphosphate
IP3	inositol-1,4,5-trisphosphate
DAG	diacylglycerol
Gi	Guanine nucleotide-binding protein, inhibitory
Gs	Guanine nucleotide-binding protein, stimulatory
TG	Thyroglobulin
TH	thyroid hormone
HPT	Hypothalamic-pituitary-thyroid axis
CRHR2	Corticotropin-Releasing Hormone Receptor 2
TTR	Transthyretin
TBG	Thyroxine-binding globulin
DIO1	Type 1 Deiodinase
DIO2	Type 2 Deiodinase
THRA	Thyroid Hormone Receptor Alpha
THRB	Thyroid Hormone Receptor Beta
DIO3	Type 3 Deiodinase
T2	3,5-diiodo-L-thyronine
rT3	Reverse Triiodothyronine
THRs	Thyroid hormone receptors
α-MSH	α-melanocyte-stimulating hormone
NA	Noradrenaline
TRHR-1	Thyrotropin-Releasing Hormone Receptor 1
TSHR	Thyroid-Stimulating Hormone Receptor
MIT	Monoiodotyrosine
DIT	Diiodotyrosine
TREs	Thyroid hormone response elements
FT3	Free Triiodothyronine
MHC	Myosin Heavy Chain
AD	Adrenaline
CRHRs	Corticotropin-releasing hormone receptors
POMC	Precursor proopiomelanocortin
PC1/3	Prohormone convertase 1/3
MC2R	Melanocortin-2 receptor
MRAPs	Melanocortin receptor accessory proteins
HDAC6	Histone deacetylase 6
GRs	Glucocorticoid receptors
GCs	Circulating glucocorticoids
